# Individual Experiences in Four Cancer Patients Following Psilocybin-Assisted Psychotherapy

**DOI:** 10.3389/fphar.2018.00256

**Published:** 2018-04-03

**Authors:** Tara C. Malone, Sarah E. Mennenga, Jeffrey Guss, Samantha K. Podrebarac, Lindsey T. Owens, Anthony P. Bossis, Alexander B. Belser, Gabrielle Agin-Liebes, Michael P. Bogenschutz, Stephen Ross

**Affiliations:** ^1^Department of Psychiatry, New York University School of Medicine, New York, NY, United States; ^2^Department of Applied Psychology, New York University, New York, NY, United States; ^3^Palo Alto University, Palo Alto, CA, United States

**Keywords:** end-of-life, anxiety, depression, psilocybin, psychedelics, hallucinogen

## Abstract

A growing body of evidence shows that existential and spiritual well-being in cancer patients is associated with better medical outcomes, improved quality of life, and serves as a buffer against depression, hopelessness, and desire for hastened death. Historical and recent research suggests a role for psilocybin-assisted psychotherapy in treating cancer-related anxiety and depression. A double-blind controlled trial was performed, where 29 patients with cancer-related anxiety and depression were randomly assigned to treatment with single-dose psilocybin (0.3 mg/kg) or niacin in conjunction with psychotherapy. Previously published results of this trial demonstrated that, in conjunction with psychotherapy, moderate-dose psilocybin produced rapid, robust, and enduring anxiolytic, and anti-depressant effects. Here, we illustrate unique clinical courses described by four participants using quantitative measures of acute and persisting effects of psilocybin, anxiety, depression, quality of life, and spiritual well-being, as well as qualitative interviews, written narratives, and clinician notes. Although the content of each psilocybin-assisted experience was unique to each participant, several thematic similarities and differences across the various sessions stood out. These four participants’ personal narratives extended beyond the cancer diagnosis itself, frequently revolving around themes of self-compassion and love, acceptance of death, and memories of past trauma, though the specific details or narrative content differ substantially. The results presented here demonstrate the personalized nature of the subjective experiences elicited through treatment with psilocybin, particularly with respect to the spiritual and/or psychological needs of each patient.

## Introduction

From the early 1960s–1970s, psychedelic drug-assisted psychotherapy was researched in the United States as a treatment for cancer-related psychological and existential distress. These trials included several hundred participants and showed improvements in depression, anxiety, fear of dying, quality of life, and pain (Kast and Collins, 1964; Kast, 1966; Pahnke, 1969; Grof et al., 1973b). Building upon this research, several recently published trials examining psilocybin to treat cancer-related psychological and existential distress demonstrated rapid, substantial, and sustained improvements in cancer-related anxiety and depression, existential distress, quality of life, and orientation toward death (Grob et al., 2011; Griffiths et al., 2016; Ross et al., 2016).

To better understand participant experiences, in-depth interviews were conducted with 13 participants treated in the Ross et al. (2016) trial, revealing several common themes related to the psilocybin experience (Belser et al., 2017; Swift et al., 2017). Here, we have selected four participants from this trial (Ross et al., 2016) whose psilocybin session included several of the themes reported in the published qualitative studies of patient experiences. We demonstrate how variable and personalized participants’ psilocybin experiences were, while still representing a relatively small number of overarching themes. The case report method is unique in that it facilitates the exploration of idiographic phenomena pertaining to the explication of individual cases. It lies between the methodologies of controlled clinical trials and qualitative methods, and complements both by incorporating quantitative and qualitative information. The psychological processes described will inform the design, measures, and hypotheses of future trials. The authors sought to illustrate some of the individualized symptoms, experiences, and clinical courses that are difficult to present using traditional reporting methods. While previously published summary data from this trial demonstrate reductions in anxiety, depression, and psychosocial distress associated with death and dying, as well as common qualitative experiential themes, the current report aims to elucidate the rich complexity and personalized nature of patient responses to psilocybin-assisted psychotherapy.

## Methods

Data from this report were collected in a completed double-blind randomized controlled trial of psilocybin-assisted psychotherapy of anxiety and depression in cancer patients (see Ross et al., 2016; Supplementary Methods and **Supplementary Figure S1** for an overview of study design) and two studies utilizing qualitative analysis of interviews from a subset of participants in the main trial (see Belser et al., 2017; Swift et al., 2017 for description of emergent themes). We present quantitative as well as qualitative data collected through participant interviews, participant-completed surveys, and notes from study therapists. See Supplementary Materials for description of the quantitative measures presented.

Various demographic data, including but not limited to names, age, and type of cancer, have been obscured to preserve anonymity. The participants presented provided written informed consent for publication of these de-identified reports and were selected to demonstrate their unique experiences and because they each benefited from the treatment in different ways.

## Results

**FIGURE 1:**
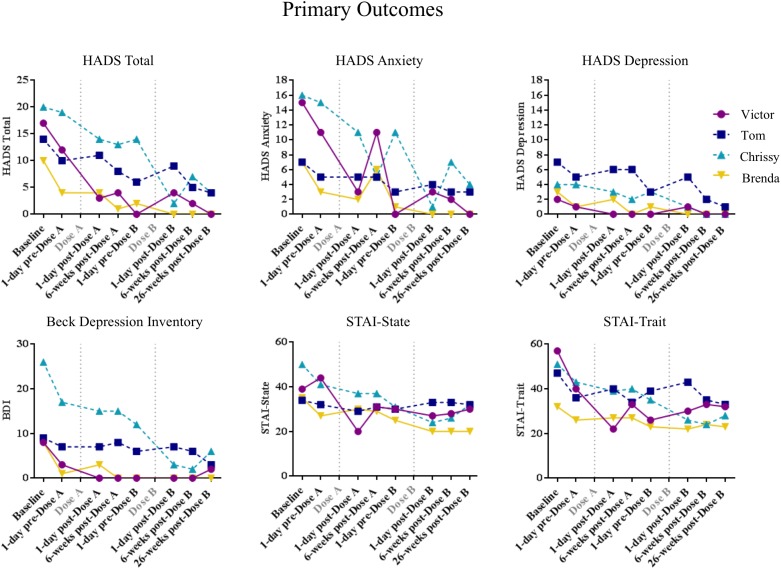
Quantitative data from the four cases presented. Scores on the Hospitalized Anxiety and Depression Scale (HADS), the Beck Depression Inventory (BDI) and the Spielberger State-Trait Anxiety Inventory (STAI) are shown here for each participant over time.

Quantitative clinical anxiety and depression results for these participants are presented in **Figure 1**, demographic information for each participant are presented in **Table 1**, and cancer-related measures of demoralization, hopelessness, and attitudes toward death are shown in **Supplementary Figure S2**. Each participant described here demonstrated improvement on multiple measures, regardless of the content of their experience.

**Table 1:** Patient demographics and pre-treatment history.

Participant	Victor	Tom	Chrissy	Brenda
Age	20s	50s	50s	60s
Gender	Male	Male	Female	Female
Race	White or Caucasian	White or Caucasian	White or Caucasian	White or Caucasian
Disease Site	Lymphoma (non-Hodgkins)	Chronic Myeloid Leukemia	Breast	Colon
Stage	IIIa	Other	IV	I
Prior Hallucinogen Use	Yes	No	Yes	No
Marital Status	Never Married	Married	Never Married	Cohabitation
Education	Completed 4-year College	Completed Grad/Professional School	Part College	Completed Grad/Professional School
Employment	Full-Time Student	Full-Time Employed	Full-Time Employed	Full-Time Employed
Religious/Spiritual Beliefs	Atheist/Agnostic	Christian	Atheist/Agnostic	Atheist/Agnostic
SCID Diagnosis	Adjustment Disorder with anxiety, chronic	Adjustment Disorder with anxiety, chronic	Generalized Anxiety Disorder	Adjustment Disorder with anxiety, chronic
Psilocybin Treatment Randomization	First Session	Second Session	Second Session	First Session


### Victor

Victor was a male in his 20s employed as a full-time graduate student at enrollment. He was raised Jewish, but renounced his faith when he was diagnosed with non-Hodgkin’s lymphoma during his sophomore year of high school. At the time of his diagnosis, Victor felt that “God had failed him,” and that his “sense of bodily invulnerability had been shattered.” When he enrolled in the trial, he was a self-described atheist and had used LSD previously. Victor was in remission at the time of enrollment but was afflicted by anxiety and fear of possible recurrence. His study therapists noted that Victor described severe anxiety, including intermittent panic attacks and constant worry about his survival. At screening, he was diagnosed with Adjustment Disorder with Anxiety, Chronic.

At the start of his psilocybin session, Victor reported seeing “geometric patterns,” with his eyes closed. He was then led on a journey by a felt presence, what he described as a “spirit guide.” “I would experience a different emotion in each part of the experience, and when that emotion became overwhelming... This spiritual guide came in through the music.” He witnessed his own conception, birth, and death, and described a vision in which he watched his family at his own funeral while feeling a “tremendously painful” helplessness. Victor noted that his session was dominated by emotional experiences and that, “whenever the affect would become overwhelming… the spirit guide would blast me out of that experience into a new setting.” Victor then said, “I didn’t have a body… I was just like this soul, this entity,” and spoke of himself shopping for a new body. The only body he could pick was his own, what he later described as a representation of the resolution of his issues with his body and illness.

I saw everything that has happened to my body, all the food I have eaten, the drugs I have taken, the alcohol [I have drunk], the people I have had sex with, the chemo, the exercise, everything that has ever happened to my body. I took it in at once, then I made this decision. Like okay, I need a body to go on, so I will choose this body. So I kind of accepted this body, and at this point I was no longer this soul spirit entity. It became me, integrated my mind into my body.

After Victor chose his body, he recalled, “there was something on top of the mountain, call it God, call it some divine entity calling me to come up this mountain… it was like a spiritual calling.” Victor asked his “spirit guide” if he could meet him. Ultimately, the “spirit guide” returned and said that God wouldn’t meet with him yet, but delivered a message that if Victor is loving and kind to other people, he might be able to meet God one day. Toward the end of the session, the “spirit guide” transformed into Victor’s father, who reassured him that everything was going to be okay. Before the end of his session, he encountered several people who he loved that had passed away, and they all shared their love for him.

His data showed decreased anxiety, and increased purpose in life, spirituality and death transcendence. In a follow-up interview, Victor stated “I would say [I have] less anxiety about my body and my sickness coming back, my cancer coming back…I saw this body for what it’s worth, I picked it, it’s mine… I think that acceptance has been liberating.” With regards to his increased spirituality, Victor stated, “I am convinced beyond any doubt that there is a spiritual realm…The spirit guide showed me a world that I believe to be very, very real.” When asked how the experience changed his attitude toward his cancer, he responded, “It is what it is…it’s not worth worrying about things you can’t change.”

### Tom

Tom was a Christian male in his 50s employed full-time in human resources upon screening. Shortly before enrolling in our trial, Tom was diagnosed with Chronic Myeloid Leukemia. At screening, he met diagnostic criteria for Adjustment Disorder with Anxiety, Chronic. Tom had never used hallucinogens at the time of enrollment.

During his psilocybin session, Tom reported seeing an inhuman, aggressive female face that he felt would bite him, given the chance. The female face transformed into a less-threatening male figure that invited him to “start” his psychedelic experience. Following this, Tom explained how the music from the study’s preselected playlist influenced his experience. “I started not just hearing, but playing the music. My entire body was the musical instrument for every sound which was coming through my head.” At one point, he removed his eye-shades to go to the bathroom and described seeing strobe-like flashing colors. He experienced visual-auditory synesthesia; he described “seeing” the music as red, blue, and green three-dimensional abstract shapes. For the next part of his experience, Tom described a sense of all-knowingness, “There is nothing to fear after you stop being in your body …it’s absolutely no hell or heaven, it’s just nothing to be afraid of.” He also detailed being surrounded by an “overwhelming feeling of love... I felt the urge to let people know to stop silly things and that nothing matters but love.” Tom described his experience as exhausting, but felt that he gained a greater appreciation for life and simultaneously lost his fear of death.

Tom showed moderately decreased anxiety and depression, hopelessness, demoralization, and death anxiety. “I don’t have a fear of death – I mean, I don’t have any desire to die…I am more interested in life now more than ever before…death in itself does not scare me,” he stated. His religiousness and spirituality data showed insignificant changes, which is illustrated qualitatively in his follow-up interview: “It was not religious in a traditional sense at all, I mean there was no religious figures.” Though Tom experienced moderate benefits in anxiety, depression, demoralization, and death anxiety, he was underwhelmed and disappointed with the psilocybin experience, its short-term effects, and its impact on his life. When asked about how his experience has affected his life, he replied, “to be honest with you, not much…I mean, it was intense, it just…was not life-changing, and I heard, for some other people, that it was.” However, despite his lackluster claim, he admitted that he discovered, “there’s nothing but love. Like the Beatles did sing, ‘All you need is love,’ that’s very true.”

### Chrissy

Chrissy was a female in her 50s, diagnosed with stage 4 breast cancer with metastases in her lungs. She was a self-described atheist and employed full-time as an administrative supervisor in the healthcare industry at baseline. She had never been married nor had children, and she lived alone. She had used both psilocybin and LSD in her past, and received a diagnosis of Generalized Anxiety Disorder upon screening.

During her qualitative interview, Chrissy said that she knew she was beginning to experience the psilocybin effects when she could “see music,” something she described as beautiful, comforting and amazing. She remembers being surrounded by the cosmos, spirits and light, and hearing words inside her head, in a voice different from her own, saying, “we are here all together,” a phrase she interpreted as welcoming her into this psilocybin-induced state. She describes a part of her experience:

I was seeing these kind of stone faces, and they were beautiful, and they would kind of come to dust, and then they would come back up, and then they would come back to dust, so I kind of think of that as like, that’s the nature of life… it rises and falls; that’s the normal way it is.

Chrissy experienced strong themes of unity and connection during this session as demonstrated by the following quote: “I felt like I could reach out to anybody and connect with them.” At one point, Chrissy saw a Ferris wheel, which she interpreted as a circle in which “life comes from death and death comes from life.” Chrissy experienced her own birth and explained, “I remember breathing, feeling my breathing, and then kind of feeling that I was coming up against a membrane of some sort. Then at some point, I came through to it, and that was just amazing.” She spoke about feeling pain in her abdomen, where her cancer was, and experienced this as her “umbilical cord to the universe.” She expressed, “this was where my life would be drained from me some day and I would surrender willingly when my time came.” Though Chrissy experienced a sense of being at peace with death, she went on to explain that she “chose to live,” and that the experience helped her reach this decision.

Chrissy experienced significantly decreased anxiety, depression, death anxiety, hopelessness, demoralization, and increased purpose in life, spirituality, and death transcendence. Chrissy said, “At one point I asked, ‘Is there going to be a cure for cancer?’ [It] doesn’t matter. We’re all going to die – doesn’t change it. That was my answer.” When prompted on a follow-up questionnaire whether her religious or spiritual beliefs had changed since her psilocybin session, she replied, “[The psilocybin experience] brought my beliefs to life, made them real, something tangible and true – it made my beliefs more than something to think about, really something to lean on and look forward to.”

### Brenda

Brenda was a female in her 60s who had stage I colon cancer, her second lifetime cancer diagnosis (she was in remission from uterine cancer) at enrollment. She was a full-time working professional and identified as an atheist at the time of her enrollment. Brenda was divorced and had two adult children. Upon screening, she identified as hallucinogen-naïve and met criteria for Adjustment Disorder with Anxiety, Chronic.

Brenda’s psilocybin experience was a “roller-coaster kind” of train ride. She described the music as being an important catalyst throughout her journey. She discussed a comforting “whirring” sound throughout the beginning of her experience that she felt was “taking her in.” At one point, Brenda felt she was contently lying on a damp cloud and thought to herself, “If this is the way it’s going to be, it’s going to be really interesting. This is going to be really amazing. And I’m ready to go.” From there, she described feeling outside of time and space, “I felt out of space and time in a way that was really, really, really comforting and beautiful.” Brenda described an experience of interconnectedness and unity, “I was the cloud, I was everything, and that was the theme throughout the whole [experience], that I was all this –this was me. And it was so wonderful… to believe that. And I still do- that is me.”

Brenda also felt as if she experienced her own death on two separate occasions during the experience and emerged both unafraid of death and viewing it as a beautiful component of existence. On the first occasion, she said, “I went into this black area and it was just wonderful... I just thought to myself... I think this might be what people experience when they die.” Her second encounter with death included seeing, “This brick thing that was a lot of bricks, and I realized this was a kind of crematorium... I was just part of this big beautiful world... and that’s what’s going to happen when I die... maybe death is a beautiful thing.”

Her experience also unearthed childhood memories of sexual assault that she realized remained unhealed. Brenda acknowledged the study as a catalyst to begin healing from this trauma. Her data depicted decreased anxiety and death anxiety. When questioned on how the session altered her life, she responded, “What’s so funny is that nobody can really see it, but yet, for me, everything has changed…I feel more contented and happy about my place in the world in all the things I’m doing.” Her data also showed an increase in spirituality, as illustrated in her follow-up interview; “So I think that’s also opened up to me tremendously – a spiritual piece. And I’ve never been religious; I’m not religious particularly at all. And I feel like I’ve really connected with a spiritual side in myself as well.” After the trial, Brenda became interested in pursuing her relationship with this new aspect of herself, and began seeking out opportunities to recollect and re-experience elements of the experience through meditation. She said:

I’ve been exploring whether I can bring back other sensations from it…I have been able to, and I’ve been doing a lot of meditating. I got into meditating afterward because it was like, ‘I just don’t want to lose this,’…I have a house up by a mountain monastery and I went up there, and that was very comforting to connect that way… I really felt like there was a real connection with Buddhism and meditation and the psilocybin experience for me. And I’ve been doing that everyday.

## Conclusion

The cases presented here were selected because their experiences were unique, but also represented several themes identified in published qualitative studies from this trial. These descriptions are not meant to be generalized. However, several broad conclusions can be drawn from these cases. Primarily, none of these participants had an experience dominated by any single theme. Rather, their experiences were rich in multiple thematic areas, while still retaining personal, meaningful, and tangible content. These four participants presented with varied psychological needs at enrollment, including symptoms of anxiety, depression, and other measures of existential distress. These distinct needs were met post-psilocybin treatment, and benefits were sustained throughout follow-up, regardless of the thematic content of their experience (**Figure 1** and **Supplementary Figure S2**).

Participants often had difficulty describing the episodic content of their medication sessions, and the emotional and cognitive impact of the experience was often easier to describe than specific content. This could be due to an inherent ineffability of the experience, or to participants’ lack of articulateness and/or vocabulary. Regardless, whether or not a psychedelic experience can be verbally described does not seem to predict its meaningfulness or clinical impact. In fact, descriptions of the psychedelic experience were frequently given in terms of how it made participants feel and how it restructured their thinking and emotional responses in everyday life, which may be more important for persisting benefit than any specific content. While visual/auditory alterations have not been demonstrated to predict clinical change, these perceptual effects do not seem to negate the benefit of other content. Although mystical experience was found to mediate the clinical benefit reported by participants in this trial (see Supplementary Materials; Ross et al., 2016 for discussion), this does not preclude the existence of other additional mediators. The experiences described herein suggest that there may be other mediators of the therapeutic potential of psilocybin-assisted psychotherapy.

Several other questions remain unanswered and should be the focus of future trials. Participant experiences did not necessarily focus on cancer, and included salient feelings of self-compassion and love, acceptance of death, new appreciation for life, and memories of past trauma. This raises the question of whether one has to be imminently facing death to gain benefit from such treatment. Lasting behavioral changes, including eating healthier, increased exercise, and non-drug spiritual and/or meditative practices were reported by all four of the participants presented here. Whether non-drug methods of altering consciousness following psychedelic-assisted psychotherapy are helpful as an adjunct to psilocybin-induced altered states of consciousness is an important question for future studies to explore.

Not only did these experiences meet each person’s psychological needs, they also helped them understand what their needs were. Thus, one therapeutic function of psilocybin may be to assist participants in achieving insight into the cause of their distress, which is supplemented by our supportive and integrative psychotherapy treatment model. The predominant view within psychedelic research is that both psychedelic medication and psychotherapy are necessary for benefits to be reported by study participants. It is likely that the clinical benefit following treatment with psilocybin versus niacin in the current trial was a result of this drug-therapy interaction. The model employed in the current trial was most similar to “psychedelic-peak therapy” from the 1950s through 1970s (Sherwood et al., 1962; Hoffer, 1967; Pahnke et al., 1970; Grof et al., 1973a). Other models utilized in psychedelic research include the psychedelic-chemotherapy model, which used a single high-dose session of psychedelic treatment with minimal psychotherapy (e.g., Hollister et al., 1969), and the psycholytic model, which used repeated lower doses of psychedelics along with psychodynamic psychotherapy (Pahnke et al., 1970). While we have demonstrated the therapeutic value of our treatment model, future trials will be needed to evaluate comparative efficacy of the various psychotherapeutic models that have been historically used, and to answer the many remaining questions regarding optimization of psychedelic-assisted psychotherapy.

## Ethics Statement

This study was carried out in accordance with the recommendations of the institutional review board of the New York University School of Medicine (NYUSoM), with written informed consent from all subjects. All subjects gave written informed consent in accordance with the Declaration of Helsinki. The protocol was approved by the institutional review board of the NYUSoM.

## Author Contributions

SR was the principal investigator of the parent trial that served as the platform for the data collection presented in this manuscript. JG, APB, and SR acted as study therapists for the parent trial that served as the platform for the data collection presented in this manuscript. TM, AB, and GA-L acted as study coordinators and/or performed data collection for the parent trial that served as the platform for the data collection presented in this manuscript. SM, TM, SP, and LO contributed to the drafting of this manuscript. All authors contributed to the conceptualization and writing and approved the final version of this manuscript.

## Conflict of Interest Statement

The authors declare that the research was conducted in the absence of any commercial or financial relationships that could be construed as a potential conflict of interest.

## Supplementary Material

FIGURE S1 Overview of study design. Treatment components by week are depicted.

FIGURE S2 Quantitative data from the four cases presented. Scores on the Death Anxiety Scale (DAS), the Hopelessness and Anxiety Inventory (HAI), the Purpose in Life (PIL) questionnaire, the Death Transcendence Scale (DTS), the Functional Assessment of Chronic Illness Therapy-Spiritual Well-Being (FACIT-SWB) and the Spiritual Transcendence Scale (STS) are shown here for each participant over time.
