# Supplementary figures and images for: Individual Experiences in Four Cancer Patients Following Psilocybin-Assisted Psychotherapy
Source: Front Pharmacol. 2018 Apr 3;9:256. doi: 10.3389/fphar.2018.00256 (PMC5891594; doi:10.3389/fphar.2018.00256)

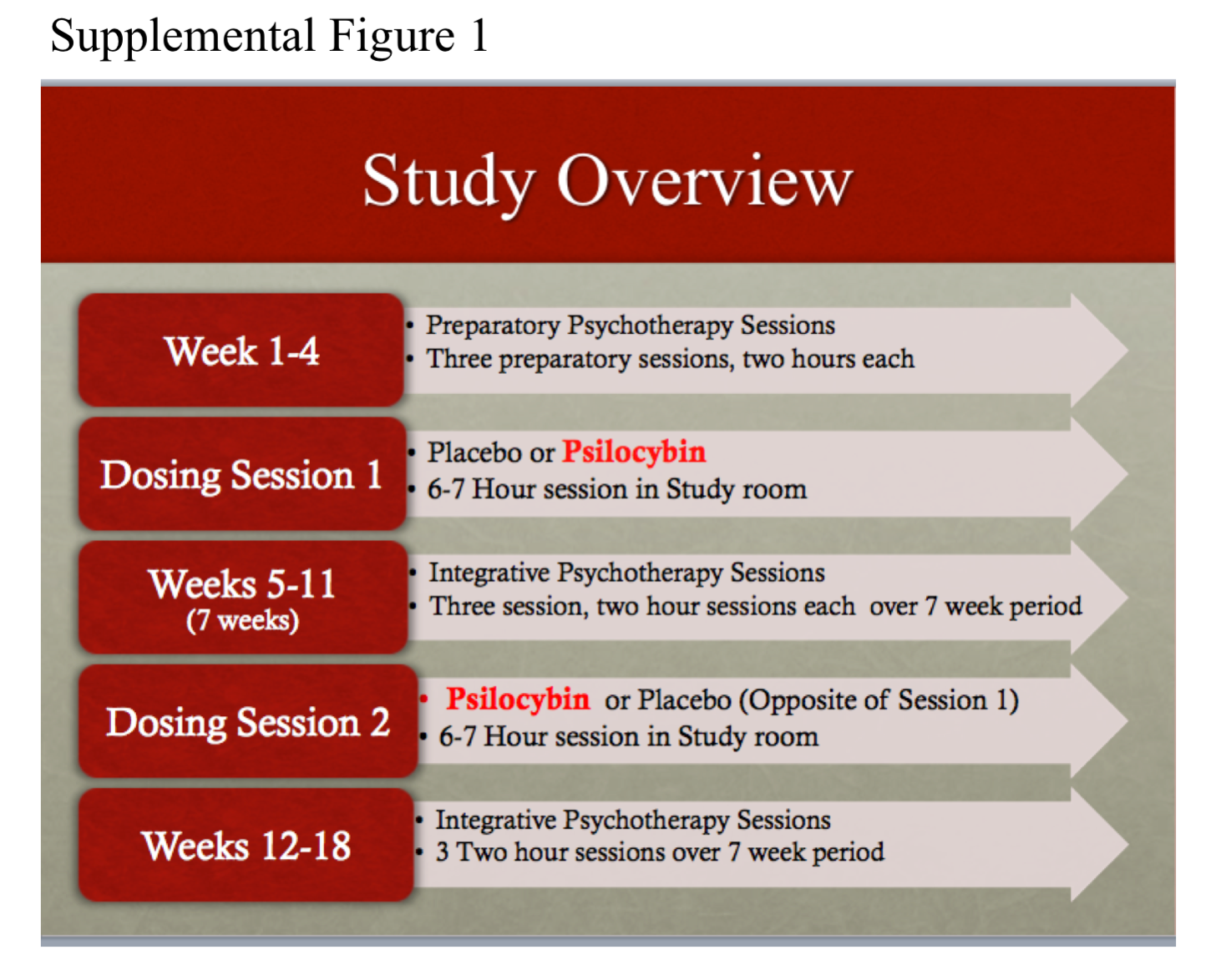

Supplement: FIGURE S1 — Overview of study design. Treatment components by week are depicted. [file Image_1.tiff]

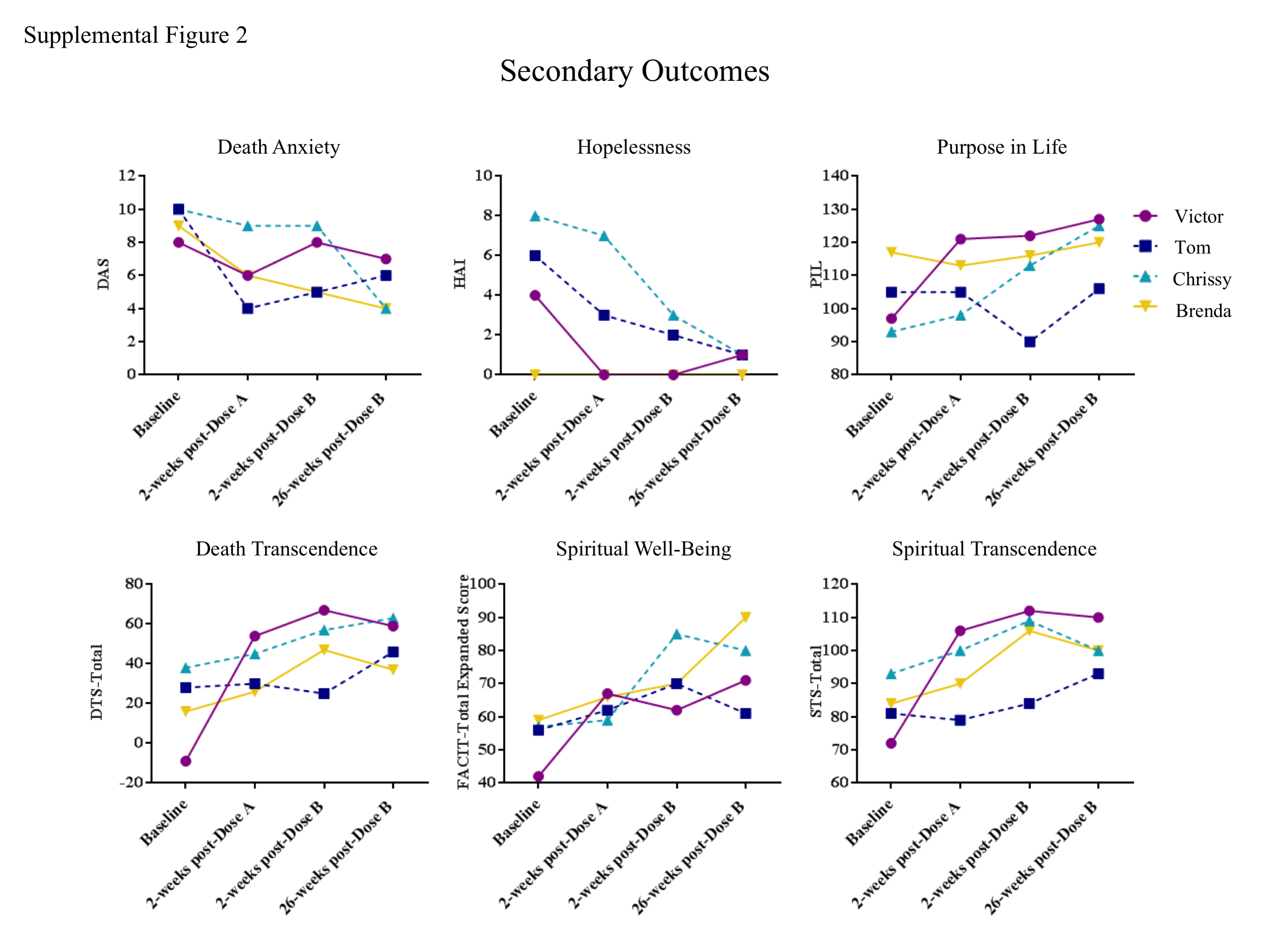

Supplement: FIGURE S2 — Quantitative data from the four cases presented. Scores on the Death Anxiety Scale (DAS), the Hopelessness and Anxiety Inventory (HAI), the Purpose in Life (PIL) questionnaire, the Death Transcendence Scale (DTS), the Functional Assessment of Chronic Illness Therapy-Spiritual Well-Being (FACIT-SWB) and the Spiritual Transcendence Scale (STS) are shown here for each participant over time. [file Image_2.tiff]
