# Supplementary material for: Individual Experiences in Four Cancer Patients Following Psilocybin-Assisted Psychotherapy
Source: Front Pharmacol. 2018 Apr 3;9:256. doi: 10.3389/fphar.2018.00256 (PMC5891594; doi:10.3389/fphar.2018.00256)
Supplement: Supplementary file 3 [file Data_Sheet_1.pdf]

Supplemental Materials

Methods

The study from which the data presented here were collected was designed to investigate the therapeutic potential of a single administration of the indolealkylamine psilocybin (0.3 mg/kg; from Organix in Woburn, MA) versus an active control (niacin 250 mg), administered alongside psychotherapy, to treat clinically significant anxiety or depression in patients with life-threatening cancer. The trial employed a two-session crossover design, with double-blind drug administration sessions occurring at approximately weeks 4 and 11 (see Ross et al., 2016 for full methods). Participants were randomly assigned to receive either psilocybin followed by niacin, or niacin followed by psilocybin.

The study employed a medication-assisted psychotherapy paradigm originally developed by Stanislav Grof that included preparatory psychotherapy prior to drug administration, support during the two 8-hour medication sessions, and integrative psychotherapy following drug administration (Grof & Halifax, 1977; Grof, Soskin, Richards, & Kurland, 1973). We chose the method of Grof et al. because this was the first and most established model of psychedelic psychotherapy, and it included the concepts of preparation, dosing, integration, set, setting, and dyadic therapy teams. Our therapeutic model was similar to the methods of Kast et al., (e.g. Kast, 1967; Kast & Collins, 1964) in several ways, despite our inclusion of extensive preparatory and integrative psychotherapy. The differences between the two approaches were chosen as an attempt to minimize anxious reactions to the psilocybin treatment and maximize the therapeutic potential of the treatment platform. Preparatory psychotherapy visits allowed participants to become familiar and comfortable with the therapy team prior to the dosing session, much like the initial observational visits described by Kast. Participants in our study were encouraged to relinquish control over the psilocybin experience, which Kast describes as likely important for attenuation of death anticipation. While we offered more extensive preparatory and integrative psychotherapy to our participants relative to Kast's work with the terminally ill, the goal of our treatment regimen was to

enhance each participant's ability to surrender to the effects of the psilocybin and minimize anxious or fearful reactions to the drug experience.

Psychedelic Therapy Platform used in the Cancer Anxiety Study.

This research utilized a psychotherapy platform that was developed specifically for use in this study, although it incorporated many of the traditional elements that have been present in academic psychedelic research since the 1960s. Each participant was treated by two co-therapists, usually a man and a woman. All sessions were conducted in the same room, which is outfitted to resemble a living room. An oriental rug is present, along with soft lighting, plants and books. Books on various religions and mystical traditions, meditation, mindfulness, death and dying, and also several books on art from around the world were available to participants to browse at the beginning of each medication session. These materials were selected by the study guides and were chosen to be attractive to participants from all religious denominations as well as non-religious participants. Participants spent very little time reading the material in the room; rather, the books were meant to create a living-room like aesthetic and to help participants relax and maintain focus on their intentions for the medication sessions. The therapy developed contained elements of palliative care, Viktor Frankl's logotherapy, existential therapy, and psychodynamic therapy. Supplemental figure 1 shows an overview of the study design and therapy sessions.

Historically, various therapy models have described experiences that were perceived by participants in psychedelic research studies to underlie or be required for lasting benefit to be realized following treatment with a psychedelic compound. These experiences have been described as 'peak' or 'peak-psychedelic' experiences, as well as 'mystical' experiences. Peak experiences were originally described by Abraham Maslow in 1964 as moments of ultimate happiness, fulfillment, and self-realization, and Maslow hypothesized that religious viewpoints were an impediment to peak experiences (Maslow, 1964). 'Peak-psychedelic' experiences were described throughout the 1950s-early 1970s and have a greater similarity to 'mystical' experiences, except that they tend to lack theological or religious

connotations. Peak-psychedelic experiences typically include feelings of unity/oneness with others and/or the environment, and loss of one's sense of a unique self. Mystical experiences have more recently been operationally defined as experiences high in content measured by the Mystical Experiences Questionnaire, which assesses feelings of internal and external harmony/unity, intellectual understanding of ultimate reality, sacredness, and ineffability (Barrett, Johnson, & Griffiths, 2015 2015; MacLean, Leoutsakos, Johnson, & Griffiths, 2012 & Griffiths, 2012; Pahnke, 1963; Stace, 1960). Scores on the Mystical Experiences Questionnaire from this trial have been reported, and were found to mediate between-group differences in clinical improvement of anxiety and depression in these participants (Griffiths, 2016; Ross et al., 2016). Whether scores on the Mystical Experiences Questionnaire predict therapeutic benefit within psilocybin-treated cancer patients (i.e. excluding a control group), or whether other qualities of the psilocybin experience mediated or predicted therapeutic benefit in this trial remains to be explored.

Preparatory Sessions

During the first four weeks, there were three preparatory sessions. The *first session* (two hours) was divided into two parts: the first half of the session invited the participant to tell his or her cancer story while the therapists listened. This is the first meeting of the therapists and participant, and it is hoped that a strong, warm rapport will develop among the three. The second half of the session was devoted to educating the participant about psilocybin, its effects, the types of interventions the therapists might make, and assuring the participant he/she would be carefully tended during the medication session. The *second session* was devoted to the Meaning Making Intervention, as developed by Virginia Lee (Lee, 2008). This method of life review allows the participant and therapists to get to know one another more deeply through the creation of a detailed exploration of the participant's life from birth, to the diagnosis of cancer, to the present, to the future, and finally death. This session was usually quite moving and often revealed deeply personal information about the participants' life histories. The *third session* was usually conducted the night before the first dosing session, and participants often presented with considerable

excitement mixed with anxiety. This session began with a spiritual/religious history, practices related to spirituality and/or religion, involvement with groups that have given strength and meaning, yoga and meditation history, belief in heaven and hell, and other beliefs surrounding death and/or an afterlife. In this context, the word ‘spiritual’ is used to describe beliefs about life and death that do not strictly adhere to a particular religion, such as the existence of a human spirit that is separate from the physical body, whereas religious beliefs are derived from an organized religion. There is likely a good deal of overlap in the fundamental content of spiritual and religious beliefs/practices, and we use both terms for the purpose of inclusivity. After the session, we turned to final preparations, repetition of guidelines for the sessions, discussing intentions for the session, and trying the earphones and headphones. Lastly, guidelines for arrival in the morning were reviewed.

Dosing Sessions

Dosing session began at 8:00 am with extensive clinical assessment, including blood pressure checks and pregnancy tests. The therapists and participant settled down and each stated their intentions for the day’s experience. Sometimes, a brief ritual was invoked. The participant took the psilocybin or placebo pill, lay down, and put on headphones and eyeshades. Carefully selected music played for the participant all day through the headphones, and was also played aloud into the room so the therapists could hear it. The participant was invited to go on an inner journey following the onset of the effects of the medicine. Therapists remained calm and non-directive, but aware of the participant’s response to the medication (including body language, facial expressions, any verbalizations, and signs of distress or discomfort), and intervened only when necessary. High, intense affect was not discouraged or soothed; the participant was encouraged to use the experience to explore difficult affective states, and avoid using normal psychological defenses to prevent unpleasant emotions from being felt. The therapists maintained comfort and safety during the dosing session, and did not engage in verbal therapy during the session. Thus, only supportive psychotherapy was given during the medication sessions, the purpose of which was to assist participants in surrendering to the effects of the study medication. Usually after 4-5 hours the

participant became ready to take off the eyeshades and headphones, and sometimes wanted to eat. The participant was encouraged not to start talking right away, but eventually the therapists invited participants to share a first narrative of their experience that day.

Integration Sessions

Integrative psychotherapy was offered after the medication sessions (sessions 4-9), and was designed to help participants find and communicate the personal meaning of their experiences and to relate it to the cancer-associated distress that they sought help with when they entered the study. These sessions served to support the memory and study of the psilocybin experience and to apply what was learned to day-to-day life. We encouraged participants to speak about the ways that they saw the world differently, and experienced themselves differently in it. Many themes emerged: the centrality of relationships, a feeling of connection, a deep sense of gratitude, forgiveness, renewed vitality, and self-efficacy. Listening to these expressions was key to integration, along with seeking ways to support the restoration of meaning in the participant's life. Many participants had mystical and/or peak-psychedelic experiences and were eager to discuss these. Particular types of psychedelic sessions can decrease psychological defenses, and for some, can allow significant psychodynamic exploration to occur in a short period of time. Therapists gently supported development or continuation of religious or spiritual practices, including prayer, yoga, journaling, or meditation. Finally, there was a brief termination process before work in the study ended. The termination was actively confirmed in the 8th session, then the separation was focused on during the 9th session, along with discussion of any desire for post-study contact. Contact after the end of the study was not prohibited by any means, but a continued therapy relationship was not allowed. A casual keeping-in-touch-type relationship was welcomed. However, as death approached for some participants, intense, frequent and highly emotional contact could emerge.

Quantitative Measures

Clinical outcomes were measured with the Hospital Anxiety and Depression Scale (HADS) (Zigmond & Snaith, 1983); Beck Depression Inventory (BDI) (Beck, Steer, & Garbin, 1988); and the

Spielberger State-Trait Anxiety Inventory (STAI) (Spielberger, 1983). Cancer-related measures of demoralization, hopelessness, and attitudes toward death were assessed at baseline, 2 weeks post-dose A, 2 weeks post dose B, and 26 weeks post-dose B. The cancer-related demoralization syndrome was assessed with the Demoralization scale (DEM) (Kissane et al., 2004). Hopelessness related to participants' cancer diagnosis was assessed with the Hopelessness Assessment and Illness scale (HAI) (Rosenfeld, Pessin, & Lewis, 2011). Anxiety about death and dying was assessed with the Death Anxiety Scale (DAS) (Templer, 1970). Positive attitudes and adaptations toward death were measured with the Death Transcendence Scale (DTS) (VandeCreek, 1999). Quality of life was assessed using the World Health Organization Qualify of Life scale, brief version (WHO-Bref) (Organization), 1994). Spirituality was assessed with the Functional Assessment of Chronic Illness Therapy-Spiritual Well-Being (FACIT-SWB) (Brady, Peterman, Fitchett, Mo, & Cella, 1999). For a more detailed overview of the quantitative measures presented here, see Ross et al. (2016).

Qualitative Outcomes

Semi-structured interviews were conducted with 13 of the 29 participants enrolled in the parent trial, and interpretative phenomenological analysis was used to identify a number of common themes. Ten themes emerged that were related to cancer, death/dying, and healing (anxiety and trauma related to cancer, lack of available emotional support, immersive and distressing effects of the psilocybin session, reconciliations with death, acknowledgment of cancer's place in life, emotional uncoupling from cancer, spiritual or religious interpretations, reconnection to life, reclaiming of presence, and confidence in the face of cancer recurrence; Swift et al., 2017). Additionally, seven themes more generally related to the psilocybin experience and how it impacted participants' lives were identified in at least 12 of the 13 qualitative interviews (relational embeddedness, emotional range, role of music as conveyor of experience, meaningful visual phenomena, wisdom lessons, revised life priorities, and desire to repeat the psilocybin experience; Belser et al., 2017). Detailed descriptions of these themes have been described elsewhere (Belser et al., 2017; Swift et al., 2017).

The participants whose experiences are described in the current manuscript were selected from the 13 participants that received semi-structured interviews as part of the ancillary qualitative study described above. Quotes from these participants and descriptions of their experiences in the trial were drawn from their transcribed responses. Previous publications utilized these transcribed interviews to identify and describe common and variant themes. The current manuscript aimed to supplement these reports by illustrating that multiple themes emerged within each psilocybin administration session, and that, although themes were common among participants, they were uniquely experienced through personal memories, narratives, and interpretations.

References

Barrett, F. S., Johnson, M. W., & Griffiths, R. R. (2015). Validation of the revised mystical experience questionnaire in experimental sessions with psilocybin. *J Psychopharmacol, 29*(11), 1182-1190.

Beck, A. T., Steer, R. A., & Garbin, M. G. (1988). Psychometric Properties of the Beck Depression Inventory: Twenty-Five Years of Evaluation. *Clinical Psychology Review, 8*, 77-100.

Belser, A. B., Agin-Liebes, G., Swift, T. C., Terrana, S., Devenot, N., Friedman, H. L., . . . Ross, S. (2017). Patient Experiences of Psilocybin-Assisted Psychotherapy: An Interpretative Phenomenological Analysis. *Journal of Humanistic Psychology, 57*(4), 354-388. doi:10.1177/0022167817706884

Brady, M. J., Peterman, A. H., Fitchett, G., Mo, M., & Cella, D. (1999). A case for including spirituality in quality of life measurement in oncology. *Psychooncology, 8*(5), 417-428.

Griffiths, R. (2016). A Single Dose of Psilocybin Produces Substantial and Enduring Decreases in Anxiety and Depression in Patients with a Life-Threatening Cancer Diagnosis: A Randomized Double-blind Trial *Neuropharmacology, 40*, S90-S91.

Grof, S., & Halifax, J. (1977). *The Human Encounter with Death*. New York, NY: E.P. Dutton.

Grof, S., Soskin, R. A., Richards, W. A., & Kurland, A. A. (1973). DPT as an adjunct in psychotherapy of alcoholics. *Int Pharmacopsychiatry, 8*(1), 104-115.

Kast, E. C. (1967). Attenuation of Anticipation: A Therapeutic Use of Lysergic Acid Diethylamide.

Kast, E. C., & Collins, V. J. (1964). Lysergic Acid Diethylamide as an Analgesic Agent. *Anesthesia and Analgesia Curent Researches, 43*(4), 285-291.

Kissane, D. W., Wein, S., Love, A., Lee, X. Q., Kee, P. L., & Clarke, D. M. (2004). The Demoralization Scale: a report of its development and preliminary validation. *J Palliat Care, 20*(4), 269-276.

Lee, V. (2008). The existential plight of cancer: meaning making as a concrete approach to the intangible search for meaning. *Support Care Cancer, 16*, 779-785.

MacLean, K. A., Leoutsakos, J.-M., Johnson, M. W., & Griffiths, R. R. (2012). Factor Analysis of the Mystical Experience Questionnaire: A Study of Experiences Occasioned by the Hallucinogen Psilocybin. *Journal for the Scientific Study of Religion, 51*(4), 721-737.

Maslow, A. H. (1964). *Religions, Values, and Peak-Experiences*.

Organization), W. W. H. (1994). Development of the WHOQOL: rationale and current status. *Int J Ment Health, 23*(3), 24–56.

Pahnke, W. N. (1963). *Drugs and Mysticism: An Analysis of the Relationship between Psychedelic Drugs and the Mystical Consciousness.* (Doctor of Philosophy), Harvard University,

Rosenfeld, B., Pessin, H., & Lewis, C. (2011). Assessing hopelessness in terminally ill cancer patients: development of the Hopelessness Assessment in Illness Questionnaire. *Psychol Assess, 23*(2), 325–336.

Ross, S., Bossis, A., Guss, J., Agin-Liebes, G., Malone, T., Cohen, B., . . . Schmidt, B. L. (2016). Rapid and sustained symptom reduction following psilocybin treatment for anxiety and depression in patients with life-threatening cancer: a randomized controlled trial. *J Psychopharmacol, 30*(12), 1165-1180. doi:10.1177/0269881116675512

Spielberger, C. D. (1983). *Manual for the State-Trait Anxiety Inventory (STAI)*. Palo Alto, CA: Consulting Psychologists Press.

Stace, W. T. (1960). *Mysticism and Philosophy*. New York: St. Martin's Press.

Swift, T. C., Belser, A. B., Agin-Liebes, G., Devenot, N., Terrana, S., Friedman, H. L., . . . Ross, S. (2017). Cancer at the Dinner Table: Experiences of Psilocybin-Assisted Psychotherapy for the Treatment of Cancer-Related Distress. *Journal of Humanistic Psychology, 57*(5), 488-519. doi:10.1177/0022167817715966

Templer, D. I. (1970). The construction and validation of a death anxiety scale. *J Gen Psychol, 82*, 165-177.

VandeCreek, L. (1999). The death transcendence scale. In P. C. Hill & R. W. Hood (Eds.), *Measures of religiosity* (pp. 442–445). Birmingham, AL: Religious Education Press.

Zigmond, A. S., & Snaith, R. P. (1983). The Hospital Anxiety and Depression Scale. *Acta psychiatr. scand., 67*, 361-370.
